# Emerging microalgal feed additives for ruminant production and sustainability

**DOI:** 10.1007/s44307-024-00024-w

**Published:** 2024-05-11

**Authors:** Mianmian Zhu, Stacy D. Singer, Le Luo Guan, Guanqun Chen

**Affiliations:** 1 Department of Agricultural, Food and Nutritional Science, University of Alberta, EdmontonAlberta, T6G 2P5 Canada; 2 Agriculture and Agri-Food Canada, Lethbridge Research and Development Centre, LethbridgeAlberta, T1J 4B1 Canada; 3 Faculty of Land and Food Systems, University of British Columbia, VancouverBritish Columbia, V6T 1Z4 Canada

**Keywords:** Microalgae, Feed additives, Ruminant production, Animal health, Growth performance, Methane emissions

## Abstract

The global demand for animal-derived foods has led to a substantial expansion in ruminant production, which has raised concerns regarding methane emissions. To address these challenges, microalgal species that are nutritionally-rich and contain bioactive compounds in their biomass have been explored as attractive feed additives for ruminant livestock production. In this review, we discuss the different microalgal species used for this purpose in recent studies, and review the effects of microalgal feed supplements on ruminant growth, performance, health, and product quality, as well as their potential contributions in reducing methane emissions. We also examine the potential complexities of adopting microalgae as feed additives in the ruminant industry.

## Introduction

The livestock sector plays a vital role in terms of safeguarding global food security and nutrition, and animal-derived products provide a significant proportion of protein intake (one-third) and calories (17%) worldwide, with a substantial contribution coming from ruminants (FAO 2018). Unfortunately, the ongoing rise in our global population is predicted to increase total food demand by 35% to 56% between the years 2010 and 2050 (van Dijk et al. 2021). The resulting escalation in demand for animal-derived foods will almost certainly trigger the expansion of ruminant livestock production, which, in turn, poses several environmental challenges, such as the potential for a consequent increase in methane emissions (Alexandratos et al. 2012; Odegard et al. 2014; Tilman et al. 2011; Yan et al. 2024). Given the increasing demand for animal products and a growing awareness of their nutritional value, along with the importance of environmental sustainability in livestock industries and the need to address potential health risks associated with animal production (for a review, see (Gilbert et al. 2021)), there is a dire need for the development of strategies to resolve these issues. Ideally, these solutions would simultaneously improve the competitiveness of ruminant production and mitigate adverse environmental impacts from the livestock sector. In this context, exploring alternative feed additives, such as microalgae, offers promising avenues to meet market demands while at the same time promoting environmental sustainability in livestock industries (Madeira et al. 2017).

The inclusion of microalgae in animal diets has gained widespread attention for aquaculture, as well as the production of poultry, monogastric mammals, and ruminants (El-Ghany 2020; Ma et al. 2024; Madeira et al. 2017; Martins et al. 2021). Microalgae are abundant in aquatic environments, possessing favorable characteristics such as rapid proliferation, widespread habtitats, and environmental adaptability, which make them easily accessible as natural food and feed sources for various aquatic organisms (Olabi et al. 2023; Udayan et al. 2021). In addition, certain microalgal species can rapidly synthesize and accumulate large amounts of high-quality nutritional and bioactive compounds, including lipids, proteins, carbohydrates, vitamins, carotenoids, and omega-3 fatty acids, and are thus attractive as multifaceted feed additives for animal production and health (Dineshbabu et al. 2019).

**Fig. 1:**
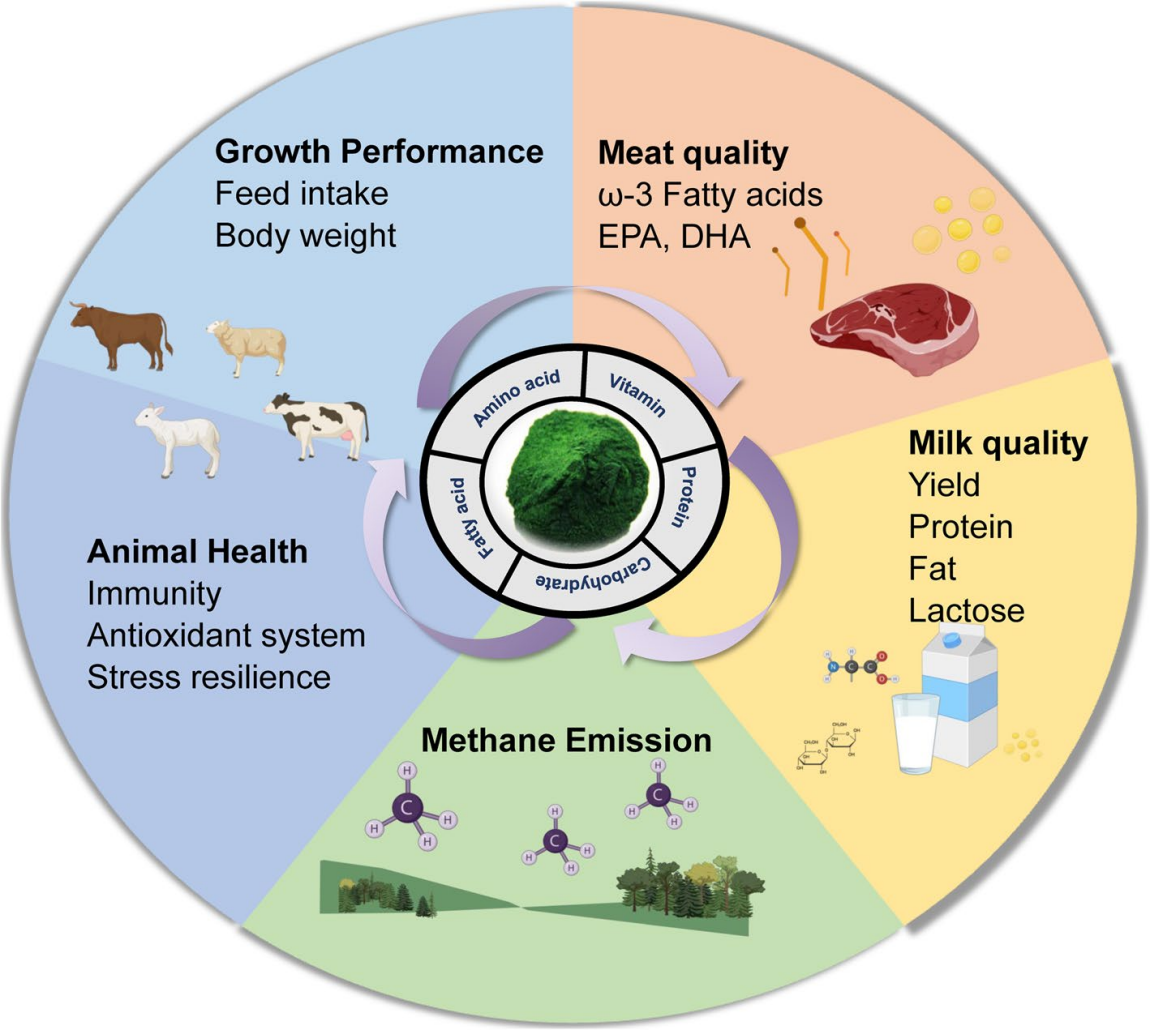
Graphical outline of effects of dietary microalgal supplementation on ruminant production and sustainability. The figure is generated in BioRender.

In this review, we discuss recent advances in the utilization of microalgal feed additives in ruminant livestock production (Fig. 1). We begin by considering the major microalgal species that have been assessed for their use as ruminant feed additives, along with their general nutritional compositions. We then discuss the influence of microalgal feed additives on the performance and health of ruminants, as well as their effects on meat and milk quality. Subsequently, we consider the influence of microalgal supplementation on ruminant methane emissions, and discuss the potential challenges and future perspectives of using microalgae as ruminant feed additives.

## Representative microalga species used as ruminant feed additives

Both prokaryotic microalgae, such as *Spirulina* (also known as *Arthrospira*), and eukaryotic microalgae, including *Chlorella* (also known as *Auxenochlorella*), *Scenedesmus*, *Schizochytrium* (also known as *Aurantiochytrium*), *Isochrysis*, *Nannochloropsis*, *Euglena*, *Micractinium*, and *Tetracystis*, have been used as ruminant feed additives to date (Table 1). Due to the rapid growth and simple structure of microalgal cells, these species can accumulate extremely high amounts of one or more of the three major nutritional components (proteins, carbohydrates, and lipids) under optimized conditions (Table 1). Among the representative species, *Spirulina* spp. and *Chlorella* spp. are the most widely utilized as ruminant feed additives, owing to their remarkable nutritional composition and the relative ease with which they can be cultivated on large-scale, industrial levels. Typically, these species can accumulate 50-70% protein on a dry weight basis with an exceptional profile of essential amino acids for ruminant dietary requirements (Anvar et al. 2021; Bito et al. 2020; Gutiérrez-Salmeán et al. 2015).

**Table 1:** General nutritional composition of microalgal species used as ruminant feed additives^a^

	% Dry weight			
Microalgae species	Proteins	Carbohydrates	Lipids	Reference
*Spirulina* spp. (*Arthrospira* spp.)	55-70	15-22	15-22	(Anvar et al.^ 2021^; Gutiérrez-Salmeán et al. 2015)
*Chlorella* spp. (*Auxenochlorella* spp*.*)	50-72	5-42	7-20	(Bito et al. 2020)
*Scenedesmus* spp.	31.6-36.2	5.3-23.6	5.3-22.8	(Amorim et al. 2020; Ji et al. 2017)
*Schizochytrium* spp. (*Aurantiochytrium* spp*.*)	7.76-21	15.19-15.23	23.86-41.2	(Hien et al. 2022; Xu et al. 2021)
*Isochrysis* spp.	41-45.3	20.6-57.2	29.6-60.3	(Valenzuela-Espinoza et al. 2002; Mohy El-Din 2019; Nalder et al. 2015)
*Nannochloropsis* spp.	22.2-37.4	28.7-40.4	15.1-60.35	(Ma et al. 2014; Rebolloso-Fuentes et al. 2001)
*Euglena* spp.	22-56	10-48 ^b^	13-14	(Nur et al. 2023; Yan et al. 2023)
*Micractinium reisseri*	15.2	31.5	33.8	(Anele et al. 2016)
*Tetracystis* sp.	13.7	28.9	37.7	(Anele et al. 2016)

^a^The composition and content of nutritional compounds in the same algal species may substantially vary according to strains, cultural conditions, and growth stages

^b^Paramylon content

The attractiveness of using microalgae as feed additives in ruminant diets also stems from their high contents of bioactive compounds in their biomass, including unsaturated fatty acids and essential vitamins, which play important roles in ruminant health and product quality. For example, *Spirulina* spp. and *Chlorella* spp. can produce high amounts of ω-6 unsaturated fatty acids (Gutiérrez-Salmeán et al. 2015) and essential vitamins (Bito et al. 2020), respectively, which can substantially enhance the health and well-being of ruminants by alleviating oxidative stress and reinforcing immunity (Ampofo et al. 2022). *Nannochloropsis* spp., on the other hand, can accumulate high levels of an omega-3 very long chain fatty acid termed eicosapentaenoic acid (EPA) under stressful conditions (Ma et al. 2014; Rebolloso-Fuentes et al. 2001), while *Isochrysis galbana* and *Schizochytrium* spp. can produce high amounts of a similar fatty acid termed docosahexaenoic acid (DHA) (Hien et al. 2022; Mohy El-Din 2019; Xu et al. 2021). Both EPA and DHA can enhance animal health and the nutritional value of animal products (Barta et al. 2021).

When using microalgae as ruminant feed supplements, it should be noted that microalgal biomass and the content of nutritional and bioactive components can be substantially affected by strains, culture conditions, and growth stages. Quality control of microalgal biomass is critical in this regard. For instance, the DHA content of the *Aurantiochytrium* sp. T66 strain can be increased to 52% of cell dry weight under optimized culture conditions, but it can be much lower under unfavorable conditions (Jakobsen et al. 2008). In addition to microalgal strain selection and cultural condition optimization, traditional mutagenesis and advanced metabolic engineering can effectively improve microalgal strains, such as increasing the contents of omega3-fatty acids or proteins, to meet various nutritional demands of ruminant growth and production (Grama et al. 2022; Trovão et al. 2022). Additionally, since only a very small proportion of microalgal species have been assessed for their value as ruminant feed additives to date, and the mechanisms driving their positive roles in ruminant production and nutrition are yet to be well-characterized, there is much room for further investment in this emerging field. For instance, other microalgal species with rapid growth rate and beneficial bioactive compounds could be evaluated, and omics technologies (e.g., genomics, transcriptomics, and metabolomics) with nutritional and physiological studies could help elucidate the specific mechanisms of how the microalgal supplementation influences rumen fermentation and nutrient metabolism of ruminants.

## Influence of dietary microalgae supplementation on ruminant performance and health

### Feed intake and body weight

The effects of microalgal feed additives on ruminant growth and performance have been reported previously (Table 2). Although the direct comparison of results across different studies is difficult due to variations in the microalgal species, amount of biomass, feeding frequency, and other factors used, the results, in general, demonstrated the promise of selected microalgal species as feed supplements. Among the tested species, *Spirulina platensis*, which contains a high amount of protein, has gained widespread recognition over the past decade. For example, the inclusion of *S. platensis* in daily steer diets increased feed intake and microbial protein production efficiency, but decreased digesta retention time (Panjaitan et al. 2015). Moreover, a linear correlation was observed between the supplementation of *S. platensis-*derived nitrogen and average daily gain (Costa et al. 2016). Furthermore, the incorporation of post-lipid-extraction *S. platensis* residue into feed diets showed a positive correlation with increased organic matter intake and digestibility (Drewery et al. 2014). Taken together, high-protein *S. platensis* appears to be a promising potential alternative supplement for cattle grazing low-protein forages, which could improve feed intake and the efficiency of nutrient utilization to promote animal growth and productivity (Costa et al. 2016; Drewery et al. 2014; Panjaitan et al. 2015).

**Table 2:** Effects of dietary microalgal supplementation on ruminant growth performance and health in some representative studies

**Ruminant**	**Microalgae**	**Amount of usage**	**Main influence**	**Reference**
Steers	*Spirulina platensis*	Post-lipid-extraction algal residue at 50, 100, and 150 mg nitrogen/kg body weight / day	Increased total digestible organic matter intake and organic matter digestibility	(Drewery et al. 2014)
*Bos indicus* steers	*S. platensis*	0.5, 1.4, 2.5 and 6.1 g / kg body weight / day	Increased feed intake; decreased retention time of digesta in the rumen; increased efficiency of microbial protein production	(Panjaitan et al. 2015)
*Bos indicus* steers	*S. platensis* and *Chlorella pyrenoidosa*	4 and 4.7 g / kg body weight / day	Increased hay intake, efficiency of microbial protein production, fractional outflow rate of digesta from the rumen, concentration of NH_3_N, molar proportion of branched-chain fatty acids, and average daily gain	(Costa et al. 2016)
Angus × Simmental steers	*Aurantiochytrium limacinum (Schizochytrium limacinum)*	100 g / steer / day	Decreased dry matter intake; no difference in body weight gain	(Carvalho et al. 2018)
Male Qaidamford cattle	*Schizochytrium* sp.	100 g and 200 g / bull / day	Increased total antioxidant capacity and concentration of glutathione peroxidase	(Xu et al. 2021)
Lithuanian black and white cows in their early lactation period	*S. platensis*	200 g / cow / day	Improved body condition and fat content	(Kulpys et al. 2009)
Lithuanian black and white cows on II-III lactation	*S. platensis*	2 g / cow / day	Increased hemoglobin and erythrocytes	(Šimkus et al. 2007)
Multiparous Finnish Ayrshire cows	*S. platensis* and* Chlorella vulgaris*	Four set of combinations (per cow per day): 0.23kg *S. platensis* and 0.24kg *C. vulgaris*; 0.47kg *S. platensis* and 0.47kg *C. vulgaris*; only 0.57kg of *S. platensis*; only 1.13kg of *S. platensis*	Increased fiber and nitrogen digestibility, and ruminal NH_3_-N concentration; changed quality of dry matter intake; did not affect ruminal pH or major volatile fatty acids	(Lamminen et al. 2017)
Lactating Damascus goats	*C. vulgaris*	5 and 10 g / goat / day	Increased feed intake and apparent diet digestibility; increased concentration of total volatile fatty acids and propionic acid; increased serum glucose concentration but decreased glutamate-oxaloacetate transaminase, glutamate-pyruvate transaminase and cholesterol concentrations	(Kholif et al. 2016)
Weaner lambs	*S. platensis*	10% (w/v) / lamb / day	Increased liveweight and body condition score	(Holman et al. 2012)
Dual-purpose Australian lambs	*S. platensis*	100 mL or 200 mL of water containing 10% (w/v) of algal biomass / lamb / day	Increased liveweight and average daily liveweight gain on simulated-drought basal diets	(Holman et al. 2014)
Fattening lambs	*S. platensis*	1 g / 10 kg body weight / day	Increased liveweight, daily live weight gain, feed intake and feed conversion ratio; increased hemoglobin, total white blood cell count, serum globulin, vitamin A	(El-Sabagh et al. 2014)
Hu lambs	*S. platensis*	3% on a dry matter basis in high energy diet / lamb / day	Improved antioxidant capacity and immunity-related parameters; ameliorated lipid metabolic disorder and oxidative stress	(Liang et al. 2020)
Canadian Arcott lambs	*Schizochytrium* sp.	1, 2, or 3% on a dry matter basis / lamb / day	No effect on dry matter intake, average daily gain, or feed conversion, but increased EPA and DHA content and body wall thickness (see Table 3 for more information)	(Meale et al. 2014)

Similarly, in the case of dairy cows, the inclusion of 200 g/day of *S. platensis* in the diet of Lithuanian black and white cows during their early lactation phase over a 90-day period led to improvements in body condition (8.5-11% fatter) (Kulpys et al. 2009). Moreover, the daily inclusion of *S. platensis* in the diets of Multiparous Finnish Ayrshire cows increased fiber and nitrogen digestibility (Lamminen et al. 2017); however, feed intake was not affected, which could be attributed to variations in the individual adaptability of cows to diets containing microalgal supplements and differences in the palatability of various doses of microalgae (Lamminen et al. 2017).

The benefits of *Spirulina* supplementation have also been reported in goats and lambs (Table 2). For instance, incorporating 10% (w/v) of *S. platensis* in daily diets over a six-week period led to an increase in the liveweight and body condition score of weaner lambs (Holman et al. 2012). Likewise, in simulated-drought basal diets, medium (100 mL) and high (200 mL) levels of 10% (w/v) of *S. platensis* supplementation per day over nine weeks resulted in an improvement in liveweight and average daily gain of White Suffolk- and Merino-sired lambs (Holman et al. 2014). In addition, the inclusion of 1 g *S. platensis* per 10 kg body weight in daily diets over 35 days not only improved live body weight and daily live weight gain, but also increased feed intake and feed conversion ratio in lambs (El-Sabagh et al. 2014).

Other microalgal species such as *Chlorella* spp. and *Schizochytrium* spp. have also been assessed in ruminant feed experiments (Table 2). While *C. pyrenoidosa* had positive effects in *B. indicus* steers (Costa et al. 2016), the incorporation of DHA-rich *Aurantiochytrium limacinum* did not show any significant influence on the body weight of ruminants (Carvalho et al. 2018; Meale et al. 2014). In addition to variations in the nutritional composition of microalgal species, this discrepancy could also be, at least in part, related to palatability and reduced feed intake (Carvalho et al. 2018). To ensure unbiased results when employing microalgae feed additives in ruminant production, it will likely be essential to evaluate the overall acceptability of the feed following the addition of microalgal species prior to feeding experiments.

### Immunity and the reduction of oxidative stress

Ruminants with robust immune and antioxidant systems can reduce susceptibility to disease and enhance adaptability to environmental stressors (Ciliberti et al. 2022). This resilience is curial for maintaining growth and animal health. Microalgae are natural sources of antioxidants and bioactive compounds that help neutralize harmful free radicals in the body, reducing oxidative stress, and mitigating inflammatory effects (Mavrommatis et al. 2023), potentially improving ruminant health and well-being. Recent studies indicated that *S. platensis* supplementation may affect physiological parameters associated with immune and antioxidant systems of ruminants and improve their health and disease resistance. For instance, the supplementation of 1 g of *S. platensis* per 10 kg body weight into the daily diets of fattening lambs resulted in elevated levels of hemoglobin, total white blood cell count, serum globulin, and vitamin A (El-Sabagh et al. 2014). Similarly, diets including the addition of 2 g of *S. platensis* biomass per day led to an 8.9% increase in the amount of hemoglobin and a 13.1% rise in erythrocytes in milk cows (Šimkus et al. 2007). Additionally, the inclusion of 3% *S. platensis* on a dry matter basis in high-energy diets over 74 days increased the activity of superoxide dismutase, total antioxidant capacity and IgG concentration in the serum of Hu lambs (Liang et al. 2020). As of yet, however, very few studies have assessed the impact of microalgal species other than *S. platensis* on ruminant immune and antioxidant systems. While one study indicated that the inclusion of 170 g and 255 g of *Schizochytrium* sp. in the diet per day over a 60-day period had no significant effect on the growth or health of dairy cows (Liu et al. 2020), another suggested that the supplementation with 100 g or 200 g of *Schizochytrium* sp. per day over 49 days enhanced the total antioxidant capacity and concentration of glutathione peroxidase in the meat of beef cattle (Xu et al. 2021), indicating that individual differences in ruminants might affect the efficacy of microalgal feed additives.

Taken together, it is clear that at least certain microalgal feed additives can improve ruminant immunity and resistance to oxidative stress, and consequently ruminant health, but further studies with different microalgal species and various ruminants are required to expand our knowledge in this area. In addition, there is also a paucity of information regarding the associated mechanisms underlying the effects of microalgal supplementation on ruminants. Recent studies have indicated that a balanced and diverse gut microbiome contributes to a well-functioning immune and antioxidant system in ruminants (Cholewinska et al. 2020; Newbold et al. 2020). Since microalgal additives in feed may provide probiotic effects on rumen microbes, it is possible that they might function to restore microbiome composition, thus alleviating inflammation and enhancing oxidative resistance (Patel et al. 2021). An extensive analysis of the dynamic interactions between microalgal feed additives and rumen microbes could help address these questions and increase our understanding of how microalgal feed additives can enhance the performance and health of ruminants.

## Influence of dietary microalgae supplementation on ruminant meat quality

Superior meat quality is characterized by taste, texture, and nutritional attributes, often warranting premium prices due to consumer preferences (Hathwar et al. 2012). As such, improving meat quality, and particularly its nutritional content, can substantially advance the profitability of ruminant production (Delgado-Pertíñez et al. 2021). Some microalgal species contain high levels of polyunsaturated fatty acids (PUFAs), vitamins, and minerals, and can be used to improve meat quality, especially PUFA contents (Table 3). *Schizochytrium* spp. stand out as a particularly intriguing microalgal species for improving meat nutritional attributes because of its high DHA content and rapid growth under heterotrophic culture in regular fermenters. Many studies have reported that feed diets supplemented with *Schizochytrium* spp. biomass led to enhanced nutritional quality in lamb muscle, characterized by a more favorable fatty acid profile and increased DHA and EPA levels (Carvalho et al. 2018; Díaz et al. 2017; Fan et al. 2019; Hopkins et al. 2014; Meale et al. 2014; Ponnampalam et al. 2016; Urrutia et al. 2016; Xu et al. 2021). In terms of other algal species, the inclusion of a dinoflagellate (Dinophyceae) in the diet of lambs also led to an increase in EPA and DHA levels in meats (Cooper et al. 2004; Elmore et al. 2005), and dietary supplementation with 4% *Isochrysis* sp. on a dry matter basis in daily diets increased the content of α-linolenic acid in lamb meat (De la Fuente-Vazquez et al. 2014).

**Table 3:** Effects of dietary microalgal supplementation on ruminant meat quality

**Ruminant**	**Microalgae**	**Amount of usage**^**a**^	**Main influence**	**Reference**
Angus × Simmental steers	*Schizochytrium limacinum (Aurantiochytrium limacinum)*	100 g / steer / day	Increased EPA, DHA and total omega-3 fatty acid content	(Carvalho et al. 2018)
			Increased lipid oxidation	
Male Qaidamford cattle	*Schizochytrium* sp.	100 g and 200 g / bull / day	Increased EPA and DHA content	(Xu et al. 2021)
Canadian Arcott lambs	*Schizochytrium* sp*.*	1, 2, or 3% on a dry matter basis / lamb / day	Increased EPA and DHA content	(Meale et al. 2014)
			Did not affect carcass characteristics	
			Increased body wall thickness	
Hu lambs	*Schizochytrium* sp*.*	3% on a dry matter basis / lamb / day	Increased EPA and DHA content	(Fan et al. 2019)
Cross ewe lambs	*Schizochytrium* sp*.*	1.8% on a dry matter basis / lamb / day	Increased EPA and DHA content in muscle	(Ponnampalam et al. 2016)
			Did not affect retail display color of fresh meat	
			Increased lipid oxidation	
Weaned male Manchego lambs	*Schizochytrium* sp*.*	2% on a dry matter basis / lamb / day	Increased DHA and total n-3 fatty acids content	(Díaz et al. 2017)
Wether lambs	*Schizochytrium* sp*.*	1.92% on a dry matter basis / lamb / day	Increased EPA and DHA content	(Hopkins et al. 2014)
			Did not affect carcass weight	
Weaned lambs	*Schizochytrium* sp.	3.89% on a dry matter basis / lamb / day	Increased DHA content	(Urrutia et al. 2016)
			Did not affect carcass traits	
			Increased lipid oxidation	
			Reduced odor and flavor ratings	
Suffolk-cross wether lambs	A Dinophyceae dinoflagellate	155 g / kg of dry matter / lamb / day	Increased EPA and DHA content in muscle and adipose tissue	(Cooper et al. 2004)
Suffolk-cross wether lambs	A Dinophyceae dinoflagellate	155 g / kg of dry matter / lamb / day	Increased EPA and DHA content in meat	(Elmore et al. 2005)
			Increased total omega-3 fatty acids content in muscle	
Weaned male lambs	*Isochrysis* sp*.*	4% on a dry matter basis / lamb / day	Increased total omega-3 fatty acids content	(De la Fuente-Vazquez et al. 2014)
			Did not affect microbial load and color characteristics	

^a^The amount of usage is presented here as “% on a dry matter basis” for comparison.

Although the inclusion of certain microalgal species in diets could increase PUFA content in meat, this dietary supplementation can also lead to reduced ratings in terms of odor and flavor (Ponnampalam et al. 2016; Urrutia et al. 2016). One possible reason for this is that high amounts of unsaturated fatty acids in ruminant meats make them more susceptible to oxidation when exposed to air, impacting overall color and flavor (Lindahl et al. 2001; Wood et al. 2008). Hence, in practical applications, careful consideration will be required to prevent potential negative impacts on ruminant meat quality, such as adopting the use of microalgae species with favorable nutritional profiles, avoiding species with pronounced and undesirable odors, and implementing strict quality control for the flavor and taste of microalgae-based additives.

## Influence of dietary microalgae supplementation on the yield and nutrition of ruminant milk

Ruminant milk is one of the most widely consumed beverages worldwide (Graulet 2014), and there is a growing interest in studying the influence of microalgal supplementation on dairy production (Table 4). Lithuanian Black-and-White cows fed forage supplemented with 2 g *S. platensis* per day over a 60-day period led to a noteworthy 7.6% or 136 kg increase in average milk production compared to a standard forage diet (Šimkus et al. 2007). Likewise, the inclusion of *S. platensis* additives in daily rations led to improved body condition and milk production in individual cows (Kulpys et al. 2009), and the supplementation with *C. vulgaris* increased the milk yield of lactating Damascus goats (Kholif et al. 2016).

**Table 4:** Effects of dietary microalgal supplementation on ruminant milk quality

**Ruminant**	**Microalgae**	**Amount of usage**	**Main influence on milk**				**Reference**
			**Yield**	**protein**	**fat**	**lactose**	
Lithuanian black and white cows on II-III lactation	*Spirulina platensis*	2 g / cow / day	Increased	Increased	Increased	Increased	(Šimkus et al. 2007)
Lithuanian black and white cows in their early lactation period	*S. platensis*	200 g / cow / day	Increased	Increased	Decreased	Increased	(Kulpys et al. 2009)
Multiparous Finnish Ayrshire cows	*S. platensis* and *Chlorella vulgaris*	0.23kg and 0.24kg; 0.47kg and 0.47kg; only 0.57kg of *Spirulina*; only 1.13kg of *Spirulina* / cow / day	Unaffected	Unaffected	Unaffected	Unaffected	(Lamminen et al. 2017)
Lactating Chinese-Holstein dairy cows	*Schizochytrium* sp.	170, and 255 g / cow / day	Unaffected	Unaffected	Unaffected, increased DHA and omega-3 fatty acids	Unaffected	(Liu et al. 2020)
Primiparous Brown Swiss and multiparous Holsteins in mid lactation	*Schizochytrium* sp*.*	910 g / cow / day	Unaffected	Unaffected	Decreased fat; increased conjugated linoleic acid, DHA and transvaccenic acid	N/A	(Franklin et al. 1999)
Prepartal Holstein cows	*Schizochytrium* sp*.*	200 g oil / cow / day ^a^	Unaffected	Unaffected	Decreased fat; increased omega-3 fatty acids and conjugated linoleic acid	Unaffected	(Vahmani et al. 2013)
Lactating Holstein cows	*Schizochytrium* sp*.*	9.35 g / kg of dry matter intake / cow / day	Decreased	Unaffected	Decreased fat; increased DHA and conjugated linoleic acid	Unaffected	(Boeckaert et al. 2008)
Holstein cows in mid lactation	*Schizochytrium* sp*.*	125, 250 and 375 g / cow / day	Unaffected	Unaffected	Decreased fat; increased DHA and conjugated linoleic acid	Increased	(Moate et al. 2013)
Multiparous Holstein-Friesian cows	*S. platensis*	5% on a dry matter basis / cow / day	N/A	Unaffected	Decreased fat; increased omega-3 fatty acids	Unaffected	(Póti et al. 2015)
Hungarian native goats	*Chlorella kessleri*	3% on a dry matter basis / goat / day	N/A	Unaffected	Increased fat; increased omega-3 fatty acids	Unaffected	(Póti et al. 2015)
Lactating Damascus goats	*C. vulgaris*	5 and 10 g / goat / day	Increased	Unaffected	Decreased fat; increased unsaturated fatty acids	Increased	(Kholif et al. 2016)
Lactating Karagouniko ewes	*Schizochytrium* sp*.*	23.5, 47 and 94 g / ewe/ day	Unaffected	Increased	Increased fat; increased omega-3 fatty acids	N/A	(Papadopoulos et al. 2002)
Primiparous and multiparous lactating Holstein cows	*Schizochytrium* sp.	39 g / kg of dry matter / cow / day	Unaffected	Unaffected	Decreased fat; increased omega-3 fatty acids	Unaffected	(Klop et al. 2016)
Assaf ewes	*Schizochytrium* sp*.*	8 g / kg of dry matter / ewe / day	Unaffected	Unaffected	Decreased fat; increased omega-3 fatty acids and conjugated linoleic acid	Unaffected	(Bichi et al. 2013)

^a^Lipid content of algae and fish oil were measured, and 200 g lipid from different sources were added to the diet, respectively

Conversely, there have been instances where the dietary inclusion of algae did not have a significant impact on milk production. For example, basal diets supplemented with *S. platensis* or *Schizochytrium* spp. had no discernible effect on dry matter intake or milk yield in various studies (Bichi et al. 2013; Franklin et al. 1999; Klop et al. 2016; Lamminen et al. 2017; Liu et al. 2020; Moate et al. 2013; Papadopoulos et al. 2002; Vahmani et al. 2013). In contrast, diets supplemented with DHA-enriched *Schizochytrium* sp. resulted in a substantial 45% reduction in both dry matter intake and milk yield in dairy cows (Boeckaert et al. 2008). The disparities among these studies primarily stem from variations in the intake efficiency of feeds containing unpalatable microalgae. However, despite the negative impact on milk yield, *Schizochytrium* supplementation significantly increased the proportion of omega-3 fatty acids in ruminant milk (Bichi et al. 2013; Boeckaert et al. 2008; Franklin et al. 1999; Klop et al. 2016; Liu et al. 2020; Moate et al. 2013; Papadopoulos et al. 2002; Póti et al. 2015; Vahmani et al. 2013).

High levels of omega-3 fatty acids, such as DHA and EPA, in ruminant milk may provide potential benefits to improve human cardiovascular health, brain development, and immune function (Barta et al. 2021). Nevertheless, PUFAs are more susceptible to oxidation than saturated and monounsaturated fatty acids (Ponnampalam et al. 2024), and therefore increased PUFA content in milk may lead to a higher risk of lipid oxidation during storage and processing, which can negatively impact the flavor and aroma of dairy products (Ponnampalam et al. 2024). In this regard, when certain microalgal species are included in ruminant diets, it is essential to carry out extensive research in comprehensively evaluating the pros and cons, such as nutritional value, flavor, palatability, shelf life, and market acceptance of the milk and milk products.

Unlike milk yield and fatty acid composition, protein and lactose levels in ruminant milk are not significantly influenced by dietary microalgae in general (Bichi et al. 2013; Boeckaert et al. 2008; Klop et al. 2016; Lamminen et al. 2017; Liu et al. 2020; Póti et al. 2015; Vahmani et al. 2013), although several studies have reported an increase in protein (Kulpys et al. 2009; Papadopoulos et al. 2002; Šimkus et al. 2007) or lactose content (Kholif et al. 2016; Kulpys et al. 2009; Šimkus et al. 2007). Taken together, these findings suggest that the inclusion of protein-rich microalgae can enhance milk production without affecting feed intake in general, while the addition of DHA-rich microalgal biomass to feed can elevate the content of omega-3 fatty acids in milk, albeit with a negative effect on feed intake due to decreased palatability. Since palatability is an important issue, it may be necessary to refine the sensory properties of at least certain microalgae feed additives to ensure their effective application.

It has been suggested that the effect of microalgal feed additives on milk production may be determined predominantly by alterations in feed intake, digestion efficiency, and rumen fermentation, which are critical for ruminant growth (Kholif et al. 2016; Kulpys et al. 2009). In terms of milk composition, modifications might possibly stem from the influence of microalgal supplementation on rumen biohydrogenation and milk synthesis. During rumen biohydrogenation, microbes convert unsaturated fatty acids such as linoleic acid and linolenic acid to saturated fatty acids, which are then incorporated into milk production (Harvatine et al. 2009; Kholif et al. 2016). Supplementation with PUFA-rich microalgal biomass has been proposed to potentially impede the hydrogenation of unsaturated fatty acids in the rumen, but at the same time also provide more conjugated linoleic acid, EPA and DHA into milk production (Kholif et al. 2016; Lourenco et al. 2010). However, further research is needed to expand our understanding of rumen biohydrogenation, as well as the influence of microalgal supplementation on this intricate process, to unravel these mechanisms in full.

## Influence of dietary microalgae supplementation on ruminant methane emissions

The livestock industry contributes a substantial proportion of greenhouse gas emissions, especially methane released during the digestive processes of ruminants (Yan et al. 2024). The potential value of dietary microalgae supplementation on the reduction of methane emissions have been studied using different microalgal species, mainly through *in vitro* assays (Table 5), and at least in certain cases the results have indicated a positive effect. For instance, adding 100 g of *Euglena gracilis* per kg of feed dry matter led to a noteworthy 9.1% reduction in methane production from non-lactating Holstein cows’ rumen fluid without adverse effects on the *in vitro* fermentation profile (Aemiro et al. 2016). Similarly, the incorporation of 5% *Nannochloropsis oculate* in Barki sheeps’ rumen fluid also reduced methane production (Gomaa et al. 2018). Moreover, diets containing post-lipid-extraction *Scenedesmus* sp. biomass significantly reduced methane production, while whole cells did not have the same effect (Tibbetts et al. 2017). Additionally, the inclusion of *Chlorella* spp. at 25% of the total incubated dry matter in the feed also resulted in a slight decrease in methane production (Sucu 2020).

**Table 5:** Effects of dietary microalgae supplementation on ruminant methane emissions

**Rumen fluid donors**	**Microalgae**	**Amount of usage**	**Main influence**		**Reference**
			**Methane Production**	**Other notable influences**	
Non-lactating Holstein cows	*Euglena gracilis*	50, 100, 200, 400 and 1000 g / kg of dry matter	Decreased	Decreased total volatile fatty acid concentration	(Aemiro et al. 2016)
				Improved dry matter digestibility	
				Decreased protozoa population	
Barki sheeps	*Nannochloropsis oculata*	1, 2, 3, 4 and 5% on a dry matter basis	Decreased	Increased gas production rate and lag time	(Gomaa et al. 2018)
Polish Holstein- Friesian dairy cows	*Nannochloropsis limnetica*	2, 4, 6% on a dry matter basis	Unaffected	Increased propionic acid concentration	(Marrez et al. 2017)
				Decreased bacteria count	
				Did not affect total volatile fatty acid concentration	
Mid-lactation Holstein-Friesian dairy cows	*Scenedesmus* sp.	Algae biomass after lipid extraction (equivalent to 32% of the total diet on a dry matter basis)	Decreased	Did not affect dry matter digestibility and apparent metabolizable energy content	(Tibbetts et al. 2017)
About two-year-old Merino male sheeps	*Chlorella vulgaris, Chlorella variabilis* and their combination	25% on a dry matter basis	Decreased	Decreased gas production	(Sucu 2020)
				Decreased total volatile fatty acid concentration	
				Increased the level of ammonia nitrogen	
Saanen goat kids	*Schizochytrium* sp.	0, 0.4, 0.8, and 1.6 mg / mL	Unaffected	Decreased total volatile fatty acid concentration	(Ruiz-Gonzalez et al. 2018)
Brown Swiss cows	*C. vulgaris*	20, 40 and 80 mg / g of dry matter	Increased	Affected gas production and CO_2_ production	(Kholif et al. 2016)
Primiparous and multiparous lactating Holstein cows	*Schizochytrium* sp.	39 g / kg of dry matter	Unaffected CH4 production/kg of dry matter intake, but increased CH_4_ production/kg fat and protein-corrected milk	Negative effects of nitrate on apparent total-tract digestibility of nutrients were alleviated	(Klop et al. 2016)

Nevertheless, the effects of microalgal feed additives on the production of rumen-derived methane tend to vary with different microalgal strains. When the effects of *Scenedesmus* sp., *C. vulgaris*, *Nannochloris bacillaris*, *Tetracystis* sp., *Micractinium reisseri*, and *Nannochloropsis granulate* on methane production were assessed using *in vitro* assays, total methane production varied across the different microalgal species, with only *Tetracystis* sp. displaying a potential trend towards reducing the generation of methane (Anele et al. 2016). In other *in vitro* studies, the inclusion of *C. vulgaris*, *Nannochloropsis limnetica*, and *Schizochytrium* sp. did not reduce, and in some cases even increased, methane emissions (Kholif et al. 2016; Klop et al. 2016; Marrez et al. 2017; Ruiz-Gonzalez et al. 2018). Since most of these studies were carried out using *in vitro* experiments, it will be of interest to examine the value of microalgae in terms of decreasing methane emissions *in vivo* in ruminants.

It should be noted that, to date, the most effective dietary algae supplementation found to mitigate methane emissions is not microalgae, but the red macroalga *Asparagopsis*, primarily due to its natural synthesis of bromoform (Zhu et al. 2021). Bromoform can interact with vitamin B12 to influence the cobamide-dependent methyltransferase reaction, which is a pivotal step in methane production (Glasson et al. 2022; Thapa et al. 2020). Extensive research has confirmed the significant contribution of *Asparagopsis* to methane reduction in ruminants (Camer-Pesci et al. 2023). While the current microalgal studies have not yet identified species as promising as *Asparagopsis*, only a very limited number of the over 72,500 microalgae species have been investigated thus far (Grama et al. 2022). Given the shared evolution and growth habitats of microalgae and red macroalgae, it is certainly possible that microalgae with similar bromoform synthetic capabilities might be identified in the future. In addition, since the genetic engineering of certain microalgal species has been well-developed, the genetic reconstruction of bromoform synthesis in microalgae could also provide a potential means of contributing to methane reductions downstream. However, bromoform might be a suspected carcinogen (www.epa.gov) and further research is needed in this regard. Finally, the screening of microalgae-derived bioactive compounds for their ability to inhibit the activities of specific methanogenic archaea or methanogenic processes in the rumen could also open alternative avenues for decreasing ruminant-derived methane emissions through the use of microalgae-based supplements.

## Challenges for adopting microalgae as feed additives in ruminant production

While microalgae as ruminant feed additives have garnered attention due to their unique nutritional value and growth characteristics, certain challenges still need to be addressed. For example, compared with other feed sources, microalgal biomass is very expensive. This is, at least in part, due to the fact that prevailing culture methods, especially autotrophic culture, have relatively low productivity (Dębowski et al. 2020), and harvesting and dewatering processes are energy-intensive and costly (Deepa et al. 2023). Method optimization of large-scale cultivation, harvesting and dewatering, and contamination control are needed to improve commercial viability while ensuring the quantity and quality of microalgal biomass. Moreover, some microalgal species, such as *Schizochytrium* and *Chlorella*, can grow well heterotrophically using well-developed large-scale fermentation processes (for a review, see (Chen et al. 2006)), which makes them promising species for the production of microalgal feed additives. Another challenge comprises obtaining approvals for the use of microalgae in food and feed. Currently, only limited microalgal species have been approved for food use, such as *Chlorella*, *Spirulina*, and *Schizochytrium* (Niccolai et al. 2019). Obtaining regulatory approvals for microalgal feed additives can be lengthy and costly, and requires strong support from research results. As such, it is currently more attractive to develop and test microalgal species with regulatory approvals in ruminant diets to mitigate this issue. Since the supplementation of microalgae in ruminant diets may result in reduced intake due to the unfavorable flavor of feed (Carvalho et al. 2018; Meale et al. 2014), further research will also be needed to improve the palatability of microalgal feed additives to ensure effective adoption. Finally, it will also be essential to comprehensively understand the effect of various microalgal species on ruminant nutrition and health, the potential interaction between microalgae and rumen microbes, and the underlying mechanisms driving their effects.

## Conclusions and perspectives

The integration of microalgae-based feed additives holds great promise to promote the sustainability of livestock industries and improve ruminant production. The increasing recognition of the nutritional value from microalgae has sparked significant interest regarding their effects as dietary supplements for ruminants. Cumulative studies have demonstrated the unique value of microalgae as nutritional supplements, effectively meeting the dietary requisites of ruminants, improving their physiological performance, and enhancing the nutritional profile of ruminant-derived products. However, further research is needed to improve microalgal production, expand our understanding of the molecular mechanisms driving the beneficial effects of microalgal feed additives, and assess the function of microalgal feed supplementation on methane emission reductions from the livestock industry, which will facilitate the use of microalgae as ruminant feed supplements in the future.

## Data Availability

Not applicable.
